# “Not in their right mind”: the relation of psychopathology to the quantity and quality of creative thought

**DOI:** 10.3389/fpsyg.2014.00835

**Published:** 2014-07-30

**Authors:** Christopher H. Ramey, Evangelia G. Chrysikou

**Affiliations:** Department of Psychology, University of KansasLawrence, KS, USA

**Keywords:** madness, creativity, psychopathology, prefrontal cortex (PFC), hypofrontality, bipolar disorder, schizophrenia, genius

## Abstract

The empirical link between psychopathology and creativity is often correlational and fraught with suspiciously causal interpretations. In this paper, we review research in favor of the position that certain forms of psychopathology that profoundly affect the neural substrates for rule-based thought (e.g., schizophrenia, bipolar disorder) can significantly influence the quantity of creative production. Because highly productive individuals, irrespective of psychopathology, often produce work of greater quality, it seems that such an increase in the quantity of one’s output positively affects the likelihood of generating those statistically rare acts and achievements identified and celebrated as creative. We consider evidence that offers support for such a claim. In addition, we explore findings from neuroscience that can address how a neural mechanism, the flexibility of which relies on tradeoffs between rule-based (e.g., prefrontal cortex) and stimulus-based (e.g., sensorimotor cortex) brain regions, is influenced by psychopathology in ways that can alter dramatically the quantity and quality of creative output.

## INTRODUCTION

There is an undeniable empirical link between psychopathology and creativity. By its very nature, however, much of this work on the “mad genius” is correlational or otherwise methodologically compromised and still fraught with suspiciously causal interpretations (Schlesinger, 2009). In this paper, we first discuss how the persistence of an ancient historical link between creativity and psychopathology has contributed to the acceptance of recent empirical evidence at face value, despite its serious methodological shortcomings. We then review research that points to a more nuanced interpretation of these positions, namely that certain forms of psychopathology that profoundly affect the neural substrates for rule-based thought can significantly influence the quantity of creative production. We propose that psychopathology that alters prefrontal cortical filtering increases creative production and, thus, the likelihood of high-quality creative work. We argue that the link between psychopathology and creativity is indirect, such that, at least under certain circumstances, the quantity of the creative output begets its quality.

## THE HISTORICAL LINK BETWEEN CREATIVITY AND MADNESS

What is the historical and cultural link between psychopathology and creativity that it should be so pervasively held today? In Plato’s (1987; trans.) dialog, *Ion*, one is told that “[a] poet… is a light thing, and winged and holy, and cannot compose before he gets inspiration and loses control of his senses and his reason has deserted him” and further that poets can only compose “not by virtue of a skill, but by divine power” (534b–c). From this rather singular source, Western culture inherits a certain ironic awe of creative individuals. Poets (in particular) are special, set apart, and close to the divine. Unfortunately, for Plato, they are also of little use, for as much as they are sporadically close to the gods, their practice misrepresents the nature of reality (e.g., in contrast to practiced philosophy and dialectic, which allows one to glimpse beyond the world of appearances); they also fail to educate the youth morally. Regarding the latter, a poet is an emotional creature and truly only superficially knowledgeable of warfare, medicine, carpentry, etc., even though such topics might arise in their compositions; thus, if they are not experts or skilled with even these matters, how could they be consulted or trusted for matters so important and lofty as how one ought to behave or the nature of right and wrong?

What is hard to believe about still regarding the creative individual as somehow “possessed” or “inspired” (which literally suggests a vessel being “breathed into,” in+spire) is that it is based on an ancient argument that sought to privilege philosophers over poets (and rhapsodes) as authorities in the Greek world, especially in regards to a general theory of knowledge and the good (see also Plato’s *The republic*). Without such an ontological or epistemological commitment, modern creativity researchers should not be so beholden to this position. Indeed, from a modern research standpoint, studying the creative process given Plato’s account would be extremely problematic anyway because the process is essentially regarded as irrational (or non-rule-based) and—more to the point—its source problematically external to the very individual in the throes of creation. Interestingly, much of the Ancient Greek world did not hold such a “passive” view of poets; there was a comfortable overdetermination of causation in which a poem was both divine and consciously composed (see Murray, 1981). Muses did not absolve responsibility; they were the personification of inspiration, which was the purposeful use and appeal by the poet to perfectly ordinary cognitive processes like memory and knowledge, as well as processes that assisted in meeting the needs of an audience (e.g., fluency in composition or performance). There was a respectful balance between what modern researchers can regard as non-conscious productive processes and those deliberate, more controlled processes. The historical and cultural link between psychopathology and creativity is fascinating, but it was never a necessary one for either layperson or scientist. It is worthwhile to consider how research on creativity (at least with respect to its relation to psychopathology) would be different had views on “possession” been more moderate.

## THE QUANTITY OF CREATIVE PRODUCTION AND THE THIRD VARIABLE PROBLEM

Perhaps because of the persistent (though problematic) link between psychopathology and creativity described above, the more recent empirical one has been easier to establish and accept—this, despite small sample sizes, lack of generalizability, lack of statistical significance, lack of proper control groups, etc., (see Schlesinger, 2009). Andreasen (1987) famously interviewed writers at the University of Iowa Writers’ Workshop and reported that 80% had at least one episode of an affective disorder, two and half times the level of control participants, and that four times as many writers had a bipolar disorder diagnosis than control participants. This link extended to first-degree relatives, who themselves had an increased level of psychopathology. Jamison (1989) also reported high levels of diagnosis of and treatment and medicalization for affective disorders in poets and playwrights. The vast majority of participants also admitted to feelings of enthusiasm, euphoria and well-being, high energy, and fluency of thought during creative episodes, suggesting a link between hypomania specifically and creativity.

Contrary to these standard accounts suggesting that psychopathology “leads” to creativity, some have even proposed that creative work may instead precipitate the occurrence of psychopathology (which is at least a logical possibility, see Ramey and Weisberg, 2004); Kaufman and Baer (2002) have concluded that a craft and profession like poetry-writing might simply attract those predisposed to psychopathology in the first place; Ludwig (1998) has found that the psychopathology-creativity link depended on the extent to which a profession or subject matter was more formal (e.g., science) or subjective (i.e., arts); but such voices are the minority. The ultimate issue here is one of explanatory motivation. Even with essentially correlational designs, many studies’ conclusions are simply unidirectional with respect to explanation or insinuation. In fact, a third-variable problem also presents itself such that any relation between psychopathology (e.g., measured by diagnosis) and creativity (e.g., measured by the quality of a poem) could actually be accounted for by their relation to some other variable (e.g., the quantity of that which is produced, itself related to energy and motivation to produce in the first place). For example Ramey and Weisberg (2004) tested the hypothesis and posthumous diagnosis offered by McDermott (2001) that Emily Dickinson exhibited symptoms of hypomania during her lifetime and that poems written during these periods would be “more creative” than poems written during other times (presumably when she did not suffer from any mood disorder). Poems written during hypomanic years were, in fact, more likely to appear in anthologized works of poetry (a measure of creativity, or quality) than poems written during other years. This relation, however, was confounded when Dickinson’s productivity was taken into account: she also wrote *more* during her hypomanic years. When they analyzed her so-called non-mood-disordered years, the likelihood of writing a quality poem also increased in years that she was more productive. Thus, it was productivity, irrespective of psychopathology, that explained the relative creativity of her poetry (see also Simonton, 2004; for a similar link between quantity and quality). In an investigation of the relation between depression and creativity, Verhaeghen et al. (2005) concluded that rumination, or the extent to which one focuses on oneself or the causes of one’s mood, accounted for one’s creativity, not depression, and that “self-reflective rumination prepares individuals to generate a larger number of ideas” (p. 230). Many of the creative arts may, thus, function as an accommodating outlet for such self-reflection. In fact, upon closer examination, it seems that in studies of both eminent and everyday creativity, the link between psychopathology and creativity is never one of extreme, incapacitating “madness” and creativity (see Richards and Kinney, 1990). Rather, the link is between creativity and certain symptoms (e.g., of hypomania) like focused motivation and drivenness to create or achieve some goal. In fact, 90% of the writers in Jamison (1989) indicated that such moods were either integral or at least very important to their work (see also Jamison et al., 1980). These are states in which the non-pathological may also find themselves and be creative. An increase in the quantity of one’s output positively affects the likelihood of generating those statistically rare acts and achievements identified and celebrated as creative. Productivity, self-reflection, and elevated moods likely serve as reinforcers for such continued practice. What is critical, it seems, is a balance between unfettered productivity and a more controlled deliberation and evaluation of the volume of produced material. Modern research in the cognitive and brain sciences, with no overt ties to Plato, offers an account of creativity under just such a premise.

## NEURAL MECHANISMS SUPPORTING CREATIVE THOUGHT AND THE INFLUENCE OF PSYCHOPATHOLOGY

Recent neuroscience research has highlighted the potential contribution of both spontaneous and controlled processes to creative thought (Zabelina and Robinson, 2010). Coming up with novel ideas or solutions necessitates the ability to generate unexpected associations, which fosters originality and uniqueness. Generating a creative product also requires the ability to evaluate the viability and efficacy of different available possibilities, as well as an uninterrupted focus on the creative task until its completion; this latter process is generally referred to as *cognitive control*. Cognitive control underlies most aspects of higher-order cognition, from attention, language, and memory to decision-making and problem solving. This set of top-down, regulatory mechanisms is supported by the prefrontal cortex (PFC) and promotes the salience of certain bottom-up, sensory information from either the environment or the internal state of the organism toward context-appropriate responses. Likewise, access to bottom-up, sensory information that is deemed irrelevant for the task at hand is diminished or eliminated (Shimamura, 2000; Miller and Cohen, 2001). Although this process of regulatory filtering is undeniably beneficial for complex cognition, under certain circumstances exerting top-down influences might constrain or impede performance on tasks that benefit from spontaneous, bottom-up thought. This tradeoff is captured by the matched filter hypothesis (MFH) for cognitive control (Chrysikou et al., 2013b), a recent theoretical proposal that highlights potential competing interactions between prefrontal and posterior or subcortical brain systems that determine the appropriate level of cognitive control filtering over bottom-up information for optimal task performance. The MFH contends that PFC-mediated cognitive control is advantageous for explicit, rule-based tasks, involving the manipulation of information that does not exceed the representational capacity of working memory, whereas the exertion of cognitive control is counterproductive for more automatic tasks, involving information that surpasses working memory limitations. For these tasks, decreased PFC regulatory filtering and increased involvement of posterior or subcortical systems (e.g., sensorimotor cortex, basal ganglia) best supports performance (see also Thompson-Schill et al., 2009).

This proposal offers a potentially ideal explanatory framework for the neural processes involved in creative thinking generally, in addition to the likely consequences of psychopathology for creative production. It has been argued that creative thought involves a flexible modulation of cognitive control that allows the creative individual to achieve an optimal balance between spontaneous and controlled processes during the different phases of creative production (see Hélie and Sun, 2010; Zabelina and Robinson, 2010). Recent neuroscientific evidence suggests that certain data-driven creativity tasks may benefit from a state of hypofrontality, wherein limited PFC regulation and the attendant unconstrained contribution of posterior sensorimotor regions support the availability of unfiltered (low-level), raw perceptual input. For example, participants who were asked to generate an uncommon use in response to pictures of common objects while undergoing fMRI showed an increased involvement of posterior, visual object-processing regions (i.e., occipitotemporal cortex), bilaterally, but they did not show significant activity in left ventrolateral PFC regions; in contrast, participants asked to generate the common use for the same objects showed the reverse effect (Chrysikou and Thompson-Schill, 2011). What’s more, inhibiting the left inferior PFC using transcranial direct current stimulation (tDCS) increased the speed in which participants generated uncommon (but not common) uses for everyday objects, as well as the number of responses generated, whereas inhibiting the right PFC or sham stimulation did not affect performance on either task (Chrysikou et al., 2013a). Critically, patients with primary progressive aphasia, a neurodegenerative disorder that primarily affects left PFC, experience increased visual accuracy in spontaneous drawing, which was not present prior to the onset of their disease (e.g., Seeley et al., 2008; Shamay-Tsoory et al., 2011). Thus, patients diagnosed with certain neuropsychological disorders that selectively diminish PFC function exhibit increased access to bottom-up sensory information that can enhance their performance on some data-driven, higher-order cognitive tasks. Overall, in line with the MFH, a hypofrontal cognitive state can be beneficial for certain bottom-up, creative generation tasks. On the other hand, other aspects of creativity likely necessitate the contribution of top-down, PFC-guided regulatory mechanisms. For instance, evaluating the appropriateness of different novel ideas requires frontal cortex mediation to assess which solution is optimal for the task at hand (e.g., Ellamil et al., 2012). As such, creativity involves rapid shifting between a hypofrontal, generative state and a PFC-guided evaluative state, a flexible and dynamic process that likely occurs iteratively numerous times until the optimal solution to a creative task is achieved (Hélie and Sun, 2010; Chrysikou et al., 2013b).

We argue that the negotiation of the tradeoffs between rule-based and data-driven neurocognitive systems in different creativity tasks can be altered by vulnerability to certain neuropsychiatric disorders characterized by PFC hypofunction such as bipolar disorder and schizophrenia. A substantial body of work has revealed that patients with schizophrenia exhibit abnormal PFC profiles marked by either lower or inefficient frontal cortex function in response to tasks that require cognitive or affective inhibition (e.g., Perlstein et al., 2003; Koike et al., 2013; Eich et al., 2014), but not perceptual filtering (e.g., Smith et al., 2011). Furthermore, a simultaneous analysis of global anatomical and functional connectivity has revealed both lower structural connectivity and diminished coherence (i.e., either abnormally increased or decreased connections) in functional connectivity among different brain regions in patients diagnosed with schizophrenia, relative to healthy control subjects, that was predictive of symptom severity (Skudlarski et al., 2010). Similarly, patients with bipolar disorder marked by psychotic features have been shown to exhibit significant disruptions in the frontoparietal control network (e.g., Baker et al., 2014). Such neurocognitive abnormalities in these forms of psychopathology may prolong periods of hypofrontality in the patients, thus altering dramatically the quantity of creative output by increasing the generative phase of creative production. As patients periodically shift to states of higher PFC regulation (e.g., as a result of pharmacological treatment), the likelihood of encountering and identifying particularly viable, high-quality creative ideas increases, due to the overall increased volume of their creative output. We note that this model is in line with evidence suggesting higher creativity in patients with mild forms or those at risk of these disorders (e.g., Richards and Kinney, 1990; Johnson et al., 2012) and not in those diagnosed with severe cases of psychopathology characterized by very limited or non-existent regulatory function. In brief, too little PFC regulation may significantly impair the quality of creative output, whereas too much PFC regulation may limit the quantity of creative production and, as a result, also hinder the likelihood of generating an idea that would be characterized as highly creative (see also Abraham, in press; Abraham et al., 2007).

## CONCLUSION

Much of past research on the relationship between psychopathology and creativity is marred by serious methodological limitations, correlational designs, and problematically unidirectional interpretations, the prevalence of which might be attributed to the curiously persistent historical link between creativity and “madness.” Here we propose that, independent of psychopathology, highly productive individuals often produce work of greater quality. As such, an increase in the quantity of one’s output positively affects the likelihood of generating those statistically rare acts and achievements identified and celebrated as creative. We argue that creativity may depend on a dynamic filtering mechanism, the flexibility of which relies on tradeoffs between rule-based (e.g., PFC) and stimulus-based (e.g., sensorimotor or subcortical) brain regions, and which, when influenced by psychopathology, can alter dramatically the quantity—and so quality—of creative output.

## Conflict of Interest Statement

The authors declare that the research was conducted in the absence of any commercial or financial relationships that could be construed as a potential conflict of interest.
